# Biohybrid Membrane Formation by Directed Insertion of Aquaporin into a Solid-State Nanopore

**DOI:** 10.1021/acsami.2c14250

**Published:** 2022-10-16

**Authors:** François Sicard, A. Ozgur Yazaydin

**Affiliations:** Department of Chemical Engineering, University College London, WC1E 7JELondon, U.K.

**Keywords:** biohybrid nanopore, aquaporin, nanodisc, directed insertion, permeability, molecular dynamics simulation

## Abstract

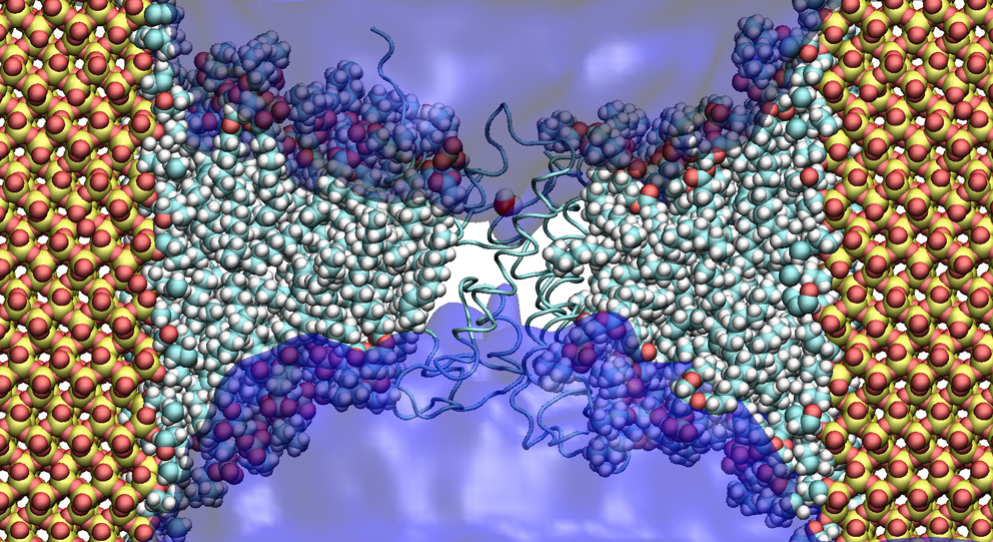

Biohybrid nanopores combine the durability of solid-state nanopores with the precise structure and function of biological nanopores. Particular care must be taken to control how biological nanopores adapt to their surroundings once they come into contact with the solid-state nanopores. Two major challenges are to precisely control this adaptability under dynamic conditions and provide predesigned functionalities that can be manipulated for engineering applications. In this work, we report on the computational design of a distinctive class of biohybrid active membrane layers, built from the directed-insertion of an aquaporin-incorporated lipid nanodisc into a model alkyl-functionalized silica pore. We show that in an aqueous environment when a pressure difference exists between the two sides of the solid-state nanopore, the preferential interactions between the hydrocarbon tail of the lipid molecules that surround the aquaporin protein and the alkyl group functionalizing the interior surface of the silica nanopore enable the insertion of the aquaporin-incorporated lipid shell into the nanopore by forcing out the water molecules. The same preferential interactions are responsible for the structural stability of the inserted aquaporin-incorporated lipid shell as well as the water sealing properties of the lipid–alkyl interface. We further show that the aquaporin protein stabilized in the alkyl-functionalized silica nanopore preserves its biological structure and function in both pure and saline water, and, remarkably, its water permeability is equal to the one measured in the biological environment. The designed biohybrid membrane could pave the way for the development of durable transformative devices for water filtration.

## Introduction

Considerable attention has been devoted to the design, characterization, and development of biohybrid nanopore-based platforms due to their potential application in the areas of environment and healthcare, including molecule sensing,^1,2^ disease diagnosis,^3^ drug design,^4^ chemical detection,^5^ pollutant removal,^6^ and water desalination.^7^ Biohybrid nanopores combine, ideally, the durability of solid-state nanopores with the precise structure and function of biological nanopores.^8^ In this perspective, tremendous progress has been made regarding the incorporation of biological compounds inside synthetic pores by modifying their surface chemistry. This includes, among others, the coating of nanopores with a supported fluid lipid bilayer^9−13^ and the insertion of biological structures in silicon-based substrates such as the α-hemolysin protein,^14^ DNA origami structures,^15^ and biological nanodiscs.^16^

We report here on the computational design of a distinctive class of biohybrid active membrane layer built from the directed insertion of an aquaporin-incorporated lipid shell in a model silica pore, which could pave the way to the development of transformative devices for water filtration. Aquaporins (Aqp) are transmembrane proteins that are present in the biological membranes of organisms such as plants, mammals, and bacteria.^17^ They form pores that act as water channels, allowing water molecules to move across the plasma membrane while rejecting protons, charged particles, and other solutes. Of particular interest is the bacterial aquaporin Z (AqpZ), expressed in *Escherichia coli*, which is the smallest, simplest, and most robust member of the Aqp family.^18^ Along with its high selectivity and water permeability, AqpZ has been purified to a high concentration, allowing it to be used for incorporation in membranes for water filtration.^10,19^

**Figure 1:**
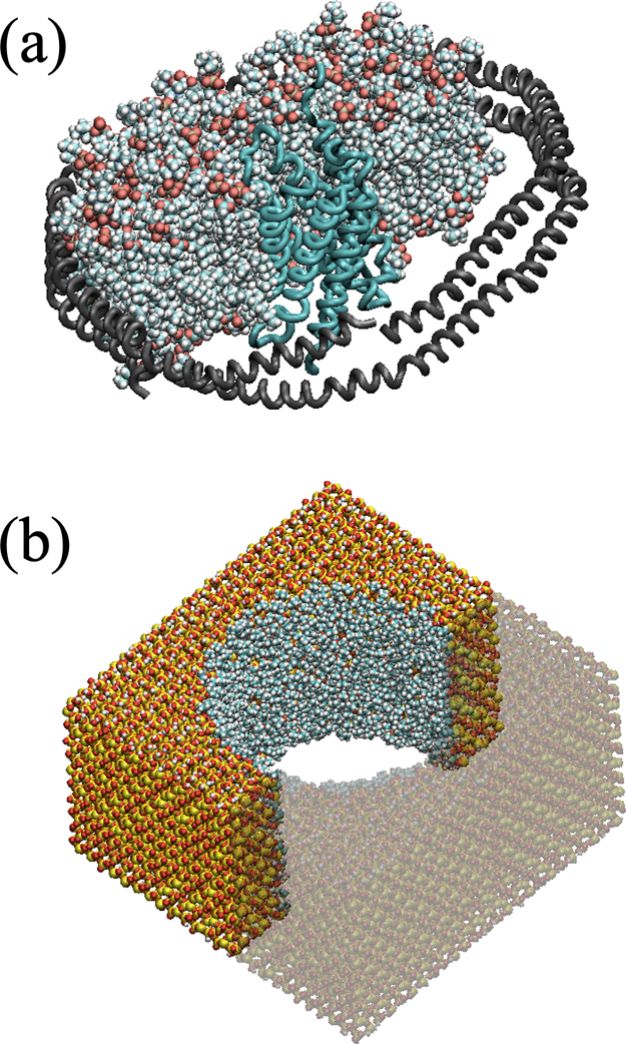
Schematic representation of (a) the AqpZ-incorporated lipid nanodisc stabilized by the MSP1E2D1 membrane scaffold protein (MSP) and (b) the cylindrical nanopore carved out of a silica-cristobalite slab. The external and internal surfaces of the slab are functionalized with hydroxyl and propyl groups, respectively. Half of the nanopore is made transparent, and half of the lipid bilayer is omitted for clarity. The backbone of the AqpZ monomer and the MSP are shown in cyan and dark gray, respectively. Yellow, red, blue, white, gold, and cyan spheres represent silicon, oxygen, nitrogen, hydrogen, phosphate, and carbon atoms, respectively.

In this work, AqpZ is originally embedded in a lipid nanodisc stabilized by membrane scaffold proteins (MSPs), as shown in Figure 1. MSPs are charged helical amphipathic proteins that wrap around the lipid bilayers with precisely defined inner and outer diameters.^20^ MSP-based lipid nanodiscs are known to provide a native membrane environment that aids in preparing integral membrane proteins in biologically active folded forms for structural studies.^21^ They have recently been shown to serve as biological templates that can be electrochemically inserted into solid-state nanopores.^16^ This integration could be a promising step toward reproducible mass production of biohybrid membranes. However, it still faces structural limitations associated with the amphipathic nature of the MSPs and their interaction with the solid-state nanopore, which affect the sealing properties of the membrane and limit its application in biohybrid nanopore technology.^16^ This is particularly important to advance the manufacturing of portable water filtration systems, for which the design of robust, thin, and defect-free membranes with high selectivity and permeability is necessary.

To circumvent this limitation, we show that, under specific conditions, the AqpZ-incorporated lipid nanodisc can be dissociated from its surrounding MSPs and inserted into a functionalized silica nanopore to form a functional biohybrid membrane layer. We first employ non-equilibrium molecular dynamics (NEMD) simulation to study the dissociation of the AqpZ-incorporated lipid nanodisc from the immobilized MSPs through the application of a pressure difference across the solid-state nanopore and its subsequent insertion into the pore. In this process, we show that the geometry of the solid-state nanopore, the nature of the functionalizing groups, and the dimension of the MSP-based lipid nanodisc can be properly chosen to ensure that the final biohybrid nanopore is stable and leakproof at the interface of the functionalized nanopore and the lipid shell. In particular, the preferential interaction between the hydrocarbon tail of the lipid molecules and the interior surface of the pore leads to the depletion of water molecules in the pore and therefore is responsible for tuning the sealing performance of the membrane. We then investigate the structural stability of the biohybrid membrane layer in pure and saline water conditions with equilibrium MD simulations. Finally, we measure the osmotic permeability of the system when a pressure difference is applied across the membrane and compare the results with those measured in the biological environment.

## Results and Discussion

### System Characteristics

We first give the characteristics of the MSP-based lipid nanodisc and solid-state nanopore considered in this work. A schematic representation of the system is shown in Figure 1. One AqpZ monomer (PDB-ID: 1RC2) is originally embedded in a 1-palmitoyl-2-oleoyl-*sn*-glycero-3-phosphocholine (POPC) lipid nanodisc stabilized by the MSP1E2D1 MSP. The diameter of the corresponding nanodisc is 11.1 nm.^22^ A model cylindrical nanopore is carved out of a silica-cristobalite slab whose surface was functionalized with hydroxyl groups (−OH). The interior of the pore is functionalized with propyl groups (−C_3_H_7_) to render its surface hydrophobic. The length of the functionalizing group corresponds to the minimal length of the carbon chain, below which adequate sealing properties of the nanopore were not achieved. The comparison with shorter groups is shown in the Supporting Information. The height and diameter of the cylindrical nanopore were specifically chosen to allow the insertion of the AqpZ-incorporated lipid shell. The latter was specifically positioned above the cylindrical pore to prevent the insertion of the MSPs into the silica pore and achieve the full depletion of water molecules. The CHARMM force field^23^ was employed to model the biological entities (MSPs, AqpZ, and POPC) and the alkyl groups on the silica surface. The INTERFACE force field^24^ and the TIP3P potential^25^ were employed to model the silica nanopore and water molecules, respectively, as they are compatible with the CHARMM force field. The simulations were conducted using the GROMACS software package, version 2018.8.^26^ The energy of the system was first optimized using the steepest descent minimization algorithm. The system was then equilibrated at a constant temperature, *T* = 300 K, finishing with equilibration at constant pressure, *P* = 1 bar, to equilibrate the water density. Finally, the production simulations were carried out in the *NVT* ensemble. NEMD simulations were performed using the extension of the pull module of the GROMACS software developed by Gräter and co-workers.^27^ The details of the numerical parameterization and the system equilibration are given in the Computational Details section and the Supporting Information, along with the molecular structure and run input files to reproduce the simulations.

### Directed Insertion of the AqpZ-Incorporated Lipid Nanodisc into a Solid-State Nanopore

**Figure 2:**
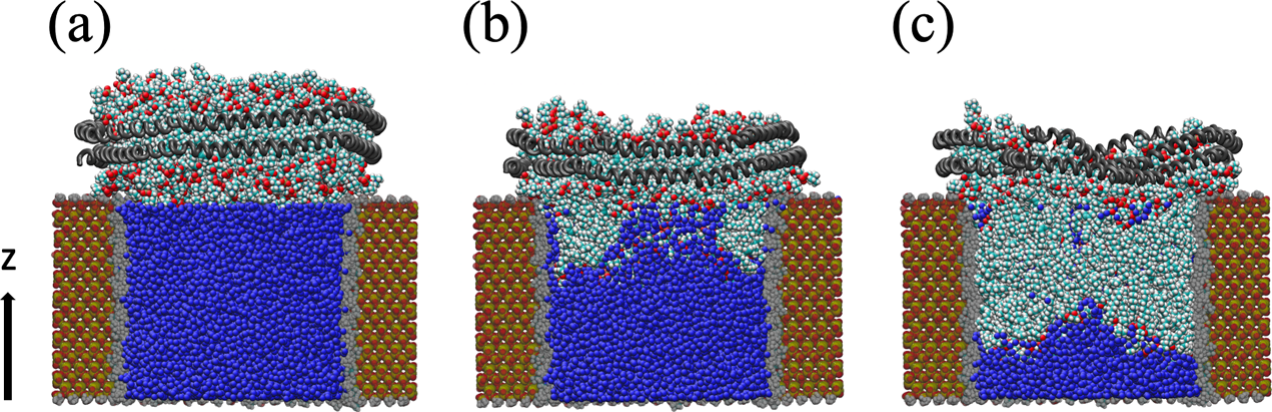
Schematic representation of the insertion of the AqpZ-incorporated lipid nanodisc into the solid-state nanopore. (a) Under the application of the hydrostatic pressure difference, the lipid nanodisc first comes in contact with the silica slab. (b) As the system moves further down the pore, the outer edge of the lipid nanodisc acts as a support layer that stabilizes the MSPs and prevents them from penetrating inside the cavity. (c) Hydrophobic tails of the lipid molecules interact favorably with the propyl groups functionalizing the interior surface of the pore, which leads to the depletion of the water molecules. The backbone of the MSPs is shown in dark gray. Yellow, red, blue, white, gold, and cyan spheres represent silicon, oxygen, nitrogen, hydrogen, phosphate, and carbon atoms in the lipid molecules and silica surface, respectively. The oxygen atoms of the water molecules are shown in blue. Only a cross-section of the pore and the water molecules in the pore are shown for clarity. A movie showing the insertion of the AqpZ-incorporated lipid nanodisc into the solid-state nanopore is provided in the Supporting Information.

To model the directed insertion of the biological membrane into the silica pore, the MSP-based lipid nanodisc is initially positioned above the cylindrical pore with its principal axis oriented in the *z*-direction orthogonal to the surface of the solid-state membrane, as shown in Figure 2. A water pressure difference is applied across the membrane to dissociate the AqpZ-incorporated lipid shell from the MSPs and to direct its insertion into the cylindrical pore. Using the numerical method of Zhu et al.,^28,29^ a pressure difference Δ*P* ∼ 80 MPa is created across the membrane and is applied for 10 ns, while the position of the solid-state membrane was restrained. The value of Δ*P* was chosen to achieve the insertion of the nanodisc in a reasonably short simulation time while preserving the structure of AqpZ, as shown in Figure S2 in the Supporting Information with the evolution of the Cartesian backbone RMSD of the protein.

Through the presence of pressure difference Δ*P*, the lipid nanodisc first comes in contact with the silica slab. Given the chosen specific orientation of the lipid nanodisc and the preferential interaction between the hydrophilic head of the lipid molecules and the hydroxyl group on the surface of the slab, the outer edge of the lipid nanodisc acts as a support layer that stabilizes the MSPs and prevents them from penetrating inside the cavity (see Figure 2a). As the AqpZ-incorporated lipid shell penetrates further into the solid-state membrane, the hydrophobic tails of the lipid molecules start interacting favorably with the propyl groups. This preferential interaction leads to the depletion of the water molecules in the pore. The depletion of water does not evenly happen around the interior surface of the pore at first, as water molecules preferentially interact with the hydrophilic head of the lipid molecules (see Figure 2b). As the simulation progresses, the AqpZ-incorporated lipid shell gradually moves further down the pore through the application of the pressure difference. As a result, the inserted lipid molecules evenly spread in the solid-state membrane, leading to the complete depletion of water around the AqpZ-incorporated lipid shell (see Figure 2c). The water pressure difference across the membrane is then switched off and the system is relaxed in a  ns simulation, which is sufficiently long to achieve the structural relaxation of the system, as shown in Figure S2 in the Supporting Information with the measure of the Cartesian backbone RMSD of the protein.

### Stability of the Active Layer in Pure Water

**Figure 3:**
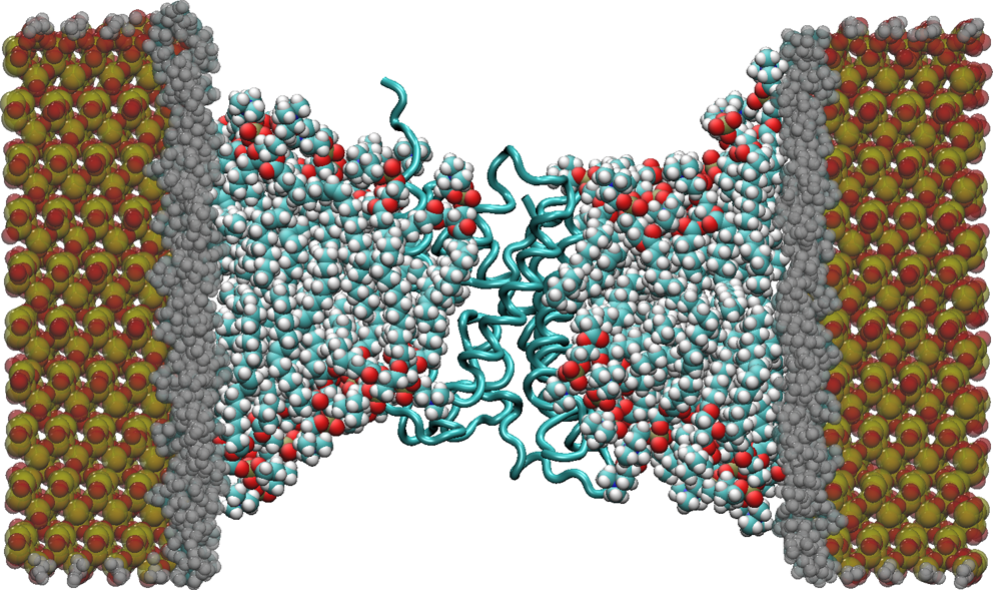
Schematic representation of the AqpZ-incorporated lipid shell inserted in the silica nanopore after the system was relaxed for 200 ns. The backbone of the AqpZ monomer is shown in cyan. Yellow, red, blue, white, gold, and cyan spheres represent silicon, oxygen, nitrogen, hydrogen, phosphate, and carbon atoms, respectively. The water molecules are not shown, and the atoms comprising the functionalized silica pore are shadowed for clarity.

**Figure 4:**
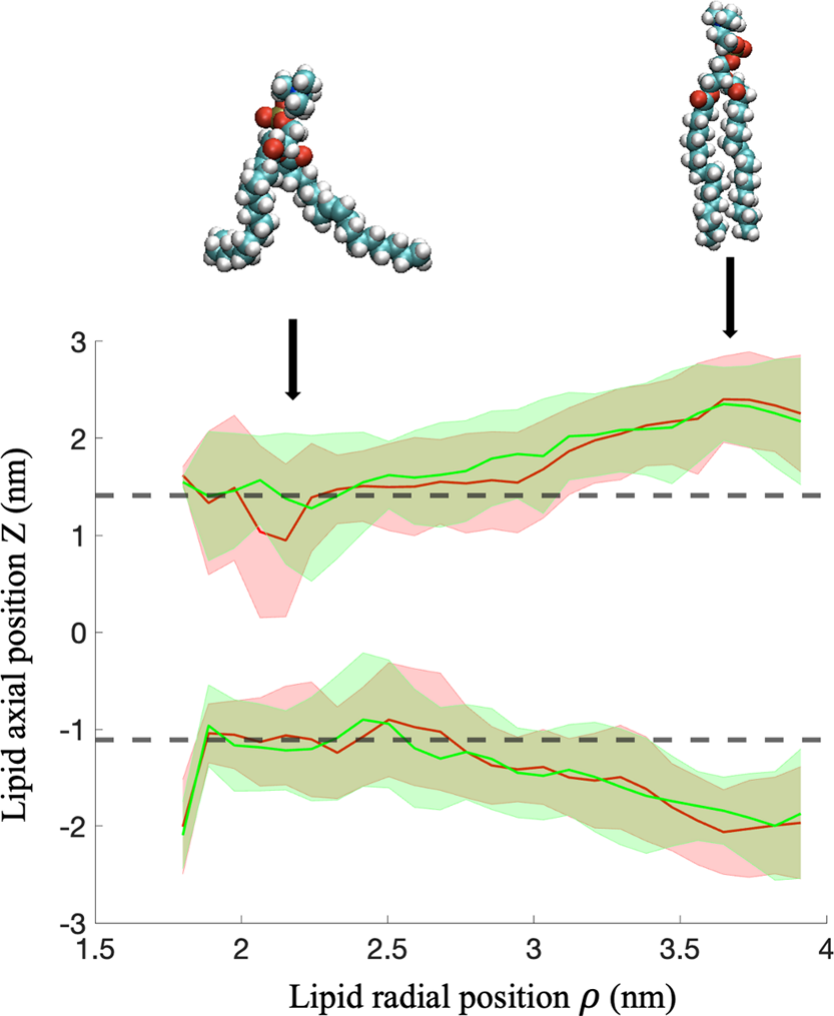
Axial position (*z*) of the lipid molecules within the ApqZ-incorporated lipid shell as a function of their radial position (ρ) in the solid-state nanopore. The cylindrical coordinates of the carbon atom *C*_2_ separating the hydrophilic head from the hydrophobic tails of the lipids (see details in the Supporting Information) are used to determine the position of the lipid molecules. The mean value of the position distribution of the lipid molecules in the biological environment is shown for comparison (dashed lines). Both the positions of the upper and lower leaflets are shown in pure (green) and saline (red) water. The thickness of the bilayer progressively increases as the lipid molecules are radially positioned away from the center of the pore. The AqpZ monomer and the interior surface of the pore are positioned at ρ < 2 nm and ρ ∼ 4 nm, respectively. Uncertainties, defined as the standard error, are represented by the shaded area.

We first study the structural stability of the biohybrid membrane layer in pure water. A representative snapshot of the system after relaxation is shown in Figure 3. We observe the increase in the thickness of the bilayer as the lipid molecules are radially positioned away from the center of the protein. This evolution is quantitatively assessed in Figure 4, where the axial position of the lipid molecules is shown as a function of their radial position with respect to the center of the pore. The average value of the position distribution of the lipid molecules measured in the biological environment, corresponding to a membrane thickness of *h*_bio_ ∼ 2.5 nm, is shown for comparison. As the lipid molecules are positioned closer to the surface of the pore, the thickness of the bilayer progressively increases, departing from the thickness measured in the biological environment. This structural deformation is associated with the configurational change of the hydrocarbon chain of the lipid molecules with a transition from a Gauche conformation near the center of the nanopore to a nearly all-trans conformation at the pore surface, as shown in Figure 4. This gauche-trans conformation transition is further assessed in Figure S3 in the Supporting Information, where the elongation along the *z*-axis of the two hydrocarbon chains forming the hydrophobic tails of the lipid molecules is shown as a function of the lipid radial position. In particular, we observe the progressive elongation of the lipid tails as the lipid radial position increases, in agreement with the increase in the membrane thickness shown in Figure 4. The gauche-trans conformation transition is due to preferential van der Waals interaction between the hydrophobic tail of lipids and the hydrophobic surface functionalized with the propyl groups.^30,31^

**Figure 5:**
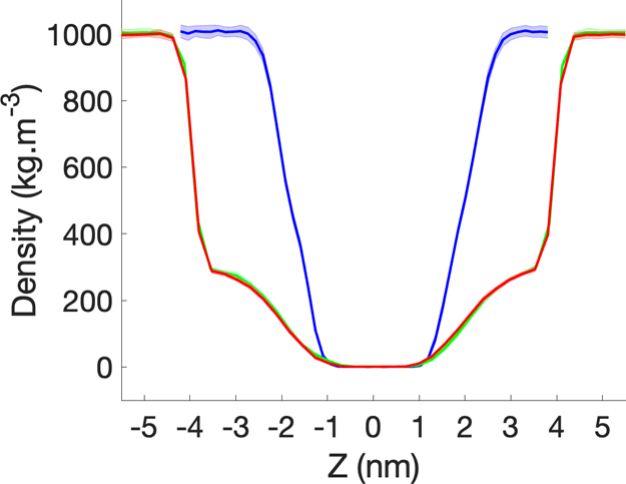
Density profile of the water molecules along the *Z* direction of the simulation box (i.e., the axis perpendicular to the membrane plane) and centered around the ApqZ monomer. Following the directed insertion of the AqpZ-incorporated nanodisc into the solid-state membrane, we let the system relax for 200 ns in pure (green) and sea saline (red) water. The density profile of the water molecules in the biological environment is shown for comparison (blue). Uncertainties, defined as the standard error, are represented by the shaded area.

To further assess the stability of the biohybrid membrane and its ability to serve as a separator, we measured in Figure 5 the density distribution of the water molecules centered around the AqpZ monomer in the simulation box after relaxation. The density distribution of water measured in the biological environment is shown for comparison. For |*z*| ≤ 1, the profile of the water density is similar to the one observed in the biological simulation. For 1 < |*z*| ≤ 4, we observe the progressive increase in the water density, which happens at a slower rate than in the biological environment. This difference is explained by the conformational organization of the lipid bilayer in the nanopore due to the preferential interaction with the hydrophobic surface, as shown in Figure 3. For |*z*| > 4, that is outside the solid-state nanopore, the system transitions abruptly to reach the bulk water density similar to the one measured in the biological environment.

### Stability of the Active Layer in Sea Saline Water

We extend the above analysis to the study of the structural stability of the active membrane layer in the presence of chloride and sodium ions at the characteristic salinity of sea water. In this condition, about 3.5% of the weight of seawater comes from dissolved salts.^32^ The evolution of the thickness of the biological membrane as a function of the radial position of the lipid molecules is shown in Figure 4. We observe an increase in the thickness of the bilayer, similar to the evolution obtained in pure water, as the lipid molecules radially position away from the center of the protein. In addition, we show in Figure 5 the density distribution of water molecules in the simulation box. The density profile is similar to the one measured in pure water. This indicates that the presence of ions does not affect the structural stability of the AqpZ-incorporated lipid shell once inserted in the solid-state nanopore. Furthermore, we computed the density distribution of ions in the simulation box after relaxation, which is shown in Figure S5a in the Supporting Information. These ions do not enter the water channel of AqpZ or the lipid shell.

This is quantitatively assessed in Figure S5b in the Supporting Information, where we measured the density distribution of ions in the simulation box. In particular, the ion density for |*z*| ≤ 1 is equal to zero. For 1 < |*z*| ≤ 4, we observe the progressive increase in the ion density due to the increase in the thickness of the biological membrane, similar to what we obtained for the density distribution of the water molecule. Finally, for |*z*| > 4, the system transitions quickly to reach the bulk ion density.

### Water Transport across the Active Membrane Layer

We complete our analysis with the study of the osmotic permeability of the biohybrid membrane layer. In the condition of reverse osmosis, the ability of the system to conduct water is characterized by the ratio of the net water flow to the hydrostatic pressure difference, Δ*P*, across the membrane. The volume flux, *J*_v_, defined as the net flow of water per unit area of the membrane is obtained as
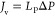
1with *L*_P_ the hydraulic permeability of the membrane.^33^ For comparison, when the two sides of the membrane have the same hydrostatic pressure but different concentrations of an impermeable solute, an osmotic pressure difference is established and water will flow from the side with the lower solute concentration to the other side. In dilute solutions, the molar water flux, *J*_m_, is linearly proportional to the solute concentration difference Δ*C*
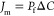
2with *P*_f_ being the osmotic permeability of the membrane.^33^ The water flux generated due to a solute concentration difference is identical to that generated by a hydrostatic pressure difference Δ*P* = *RT*Δ*C*, with *R* being the gas constant and *T* the temperature of the system. Therefore, *L*_P_ and *P*_f_ are related by a constant factor
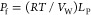
3with *V*_W_ being the molar volume of water.^28,29,33^

To determine the hydraulic permeability of the active membrane layer, a water pressure difference is applied across the membrane. The position of the membrane is restrained along the direction of the pressure gradient to avoid undesirable translation of the system. Details of the numerical implementation are given in the Computational Details section and the Supporting Information. The water flux, *J*_W_, through the channel in the membrane can then be measured at the atomistic level by counting the net number of water molecules passing through the channel during the simulation. Within the duration of the numerical simulations, no water molecules crossed through the lipid bilayer or through the interface of the lipid-propyl group functionalizing the interior surface of the silica nanopore.

**Figure 6:**
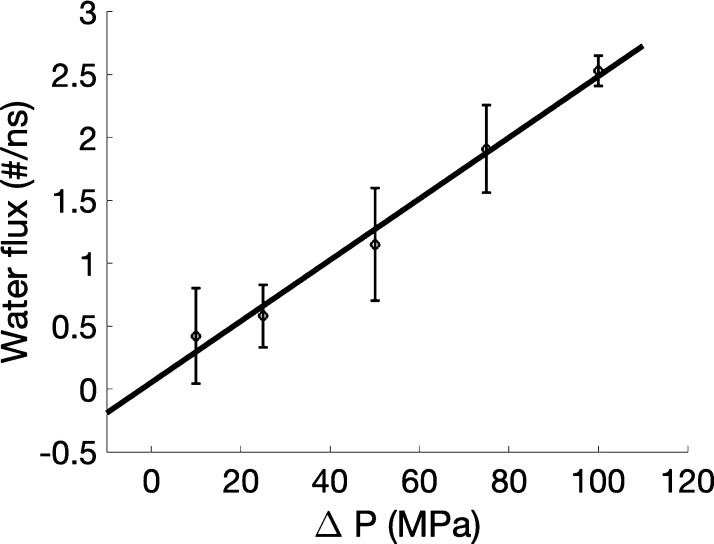
Dependence of the water flux on the applied pressure difference (10, 25, 50, 75, and 100 MPa). Error bars represent the standard deviations of the water flux. The line corresponding to the best-fit slope for the five data points is shown. Details of the simulations are given in the Supporting Information, along with a movie showing the crossing of a water molecule through the AqpZ channel.

We consider five values for the hydrostatic pressure difference, ranging from 10 to 100 MPa, as shown in Figure 6. These values are sufficiently high to assess the measure of the water flux in a reasonable simulation time without affecting the linear relationship between the water flux and the hydrostatic pressure difference.^28^ For comparison, the osmotic pressure of physiological solutions is usually below 10 MPa,^34^ whereas the osmotic pressure measured for sea water is  MPa and the pressure traditionally used in reverse osmosis desalination of sea water is  MPa.^35^

The evolution of the volume flux, *J*_v_, across the active membrane as a function of the hydrostatic pressure difference, Δ*P*, is shown in Figure 6. Each NEMD simulation was run for at least  ns, and the hydraulic permeability, *L*_P_, of the membrane is determined by interpolating the best-fit slope through the data points. Importantly, we observe that the linear relation between the water flux and the hydrostatic pressure difference, as given in eq 1, holds. We then measure the value for the hydraulic permeability, *L*_P_ = 7.3 ± 0.5 × 10^–18^ cm^5^ N^–1^ s^–1^. Applying eq 3 with *T* = 300 K, we obtain the osmotic permeability *P*_f_ = 1.1 ± 0.1 × 10^–13^ cm^3^ s^–1^. This result is in excellent agreement with the osmotic water permeability of the AqpZ monomer in biological conditions estimated from simulations^36^ and experiments,^37^ and , respectively. This result confirms that the biological function of the AqpZ monomer is preserved after its directed insertion into the silica nanopore.

## Conclusions

Whereas experimental verifications are obviously required to test our expectations, the numerical simulations discussed above allowed us to explore the design and stability of a distinctive class of biohybrid active membrane layers built from the directed insertion of an aquaporin-incorporated lipid nanodisc into a model silica nanopore. Considering the chosen specific orientation of the lipid nanodisc, with its principal axis oriented in the direction orthogonal to the surface of the solid-state membrane, we described in detail the mechanisms at play in the insertion of the biological membrane into the solid-state nanopore. We showed that the preferential interaction between the hydrophobic chain of the POPC lipid bilayer and the alkyl group functionalizing the interior surface of the pore played an essential role both in the depletion of water molecules in the pore and the sealing properties of the system. We numerically assessed the osmotic permeability of the biohybrid membrane and compared it with the experimental permeability of the AqpZ monomer in the biological environment. In particular, we showed that the biological function of the AqpZ monomer, in terms of water transport, was preserved after the directed insertion into the silica nanopore.

To further advance the design of the biohybrid membrane, it would be desirable to consider how the presence of the AqpZ tetramer, as well as different water pressure gradients and inclination angles of the MSP-based lipid nanodisc (with respect to the axis of the solid-state nanopore), would affect the directed insertion of the biological membrane into the functionalized silica pore described here. This could potentially affect the depletion of water in the pore due to the non-preferential interaction between the hydrophobic coating of the interior of the pore and the amphipathic scaffold proteins stabilizing the lipid nanodisc. Additionally, one could consider different pore geometry and functionalization groups or lipid molecules with increased rigidity, which would affect the deformation of the lipid bilayer once in contact with the interior of the pore. Whereas the diameter of the solid-state nanopore must be smaller than the size of the nanodisc to prevent the insertion of the MSPs into the pore, it could also be reduced to further tune the number of lipid molecules inserted around the protein. Due to the structural deformation of the lipid shell in contact with the interior surface of the pore, the decrease in the dimension of the pore could potentially affect the structural conformation of the protein, therefore impacting its biological function, specifically the transport of water molecules across the biohybrid nanopore. This analysis would be essential to propose an optimal design of the pore in terms of geometry and chemistry under various biological and industrial conditions, which would benefit from experimental protocols used to insert biological structures in silicon-based substrates, including concentration gradient-based and electrophoresis-driven diffusion.^14,16^ Finally, to further characterize the insertion of the nanodisc into the solid-state nanopore, it would be desirable to put into perspective our observation obtained in non-equilibrium conditions with a thermodynamic interpretation of the spontaneous insertion of the nanodisc under equilibrium conditions. In particular, López et al. showed that the presence of MSPs and transmembrane proteins can favorably influence the desorption of lipids from the nanodisc.^38^ However, the significant value of the free energy difference associated with the desorption of lipid molecules , along with the hydrophobic nature of the functionalizing group, are two limiting aspects regarding the spontaneous insertion of the nanodisc into the solid-state nanopore.

The model biohybrid nanopore designed in this work can be instrumental to the development of robust, thin, and defect-free membranes with high selectivity and permeability. Incorporating aquaporin proteins into compatible solid-state nanopores, while ensuring industrial production scalability, will be decisive for translating the nanoscale science into transformative water desalination technology. In particular, membrane technology has been dominating the water desalination industry for decades due to its high efficiency and reliability.^39^ However, the experimental performance of biomimetic desalination membranes based on the incorporation of aquaporins in polymers remains far below the performance of aquaporins in biological membranes.^40^ The incorporation of aquaporin proteins into compatible solid-state nanopores provides biohybrid technology with the potential for site-specific genetic engineering or chemical modifications, which could potentially be used as an active membrane layer in both reverse osmosis and forward osmosis applications to meet the increasing demand for fresh water at lower energy consumption and operating costs.

## Computational Details

MD simulations were performed with the GROMACS software package, version 2018.8.^26^ The CHARMM force field^23^ was employed to model the biological entities (MSP, AqpZ, and POPC) and the alkyl groups on the silica surface. The INTERFACE force field^24^ and the TIP3P potential^25^ were employed to model the silica nanopore and water molecules, respectively, as they are compatible with the CHARMM force field. The time step used in all the simulations was 0.002 ps, and the list of neighbors was updated every 0.04 ps with the grid method and a cutoff radius of 1.2 nm. The LINear Constraint Solver (LINCS) algorithm^41^ handled bond constraints, while the particle-mesh Ewald scheme^42^ was used to treat long-range electrostatic interactions. The non-bonded van der Waals cutoff radius was 1.2 nm. The initial velocities were chosen randomly. The cutoff algorithm was applied for the non-Coulomb potentials with a radius of 1.2 nm.

The energy of the system was first optimized using the steepest descent minimization algorithm. The system was then equilibrated for 250 ps at constant temperature, *T* = 300 K, using the Berendsen thermostat with lower restraints. The equilibration phase was then conducted within the isobaric-isothermal (*NPT*) ensemble to equilibrate the fluid density. The temperature and pressure were maintained at *T* = 300 K and *P* = 1 bar, respectively, using the Berendsen thermostat and barostat for 2 ns. We then switched to the Nose–Hoover thermostat and the Parrinello–Rahman barostat for an additional 2 ns, which are considered more thermodynamically consistent algorithms. Finally, the simulation was continued in *NVT* conditions, coupling with the v-rescale thermostat at constant temperature *T* = 300 K. We let the system run for an additional 10 ns to generate two additional initial configurations, which allowed us to test the reproducibility of the simulations.

**Figure 7:**
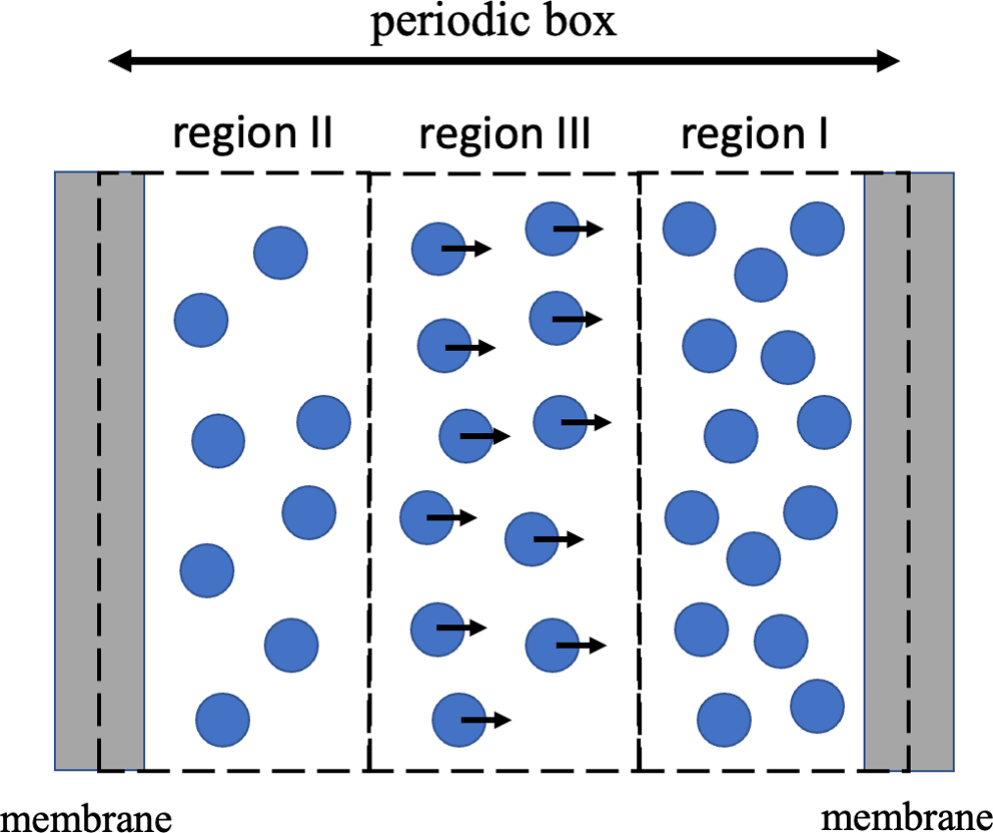
Schematic representation of the numerical method implemented to produce a pressure difference in MD simulations using periodic boundary conditions. A constant force *f* is exerted on the oxygen atoms of the water molecules in region III. The membranes shown in the figure are the periodic images of each other.

To achieve the insertion of the AqpZ-incorporated lipid nanodisc into the solid-state nanopore and to measure the osmotic permeability of the system, a hydrostatic pressure difference was generated across the membrane using the method proposed by Zhu et al.^28,29^ and the extension of the pull module of the GROMACS software developed by Gräter and co-workers.^27^ As shown in Figure 7, three regions (I, II, and III) were defined in the water layer, with region III isolated from the two sides of the membrane by regions I and II, respectively. A constant force *f* along the *z*-direction (orthogonal to the plane of the membrane) is exerted on all water molecules in region III, generating a pressure difference between regions I and II
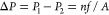
where *n* is the number of water molecules in region III, and *A* is the area of the membrane. Given the thickness of region III, *d*, the number of water molecules in this region is *n* = *Ad*/*v*_W_, with *v*_W_ the average volume of a single water molecule. Therefore, we obtain the chemical potential difference of water between regions I and II, Δμ = *fd*, associated with the pressure difference Δ*P* = Δμ/*v*_W_ between these 2 regions,^33^ which results in a net water flow through the channel. To prevent the overall translation of the whole system along the direction of the applied forces, position constraints were applied to the membrane in the *z*-direction.^29^ In particular, we applied the constant force (*f*) on the oxygen atoms of the water molecules in region III, defined as a 8 nm thick layer. When necessary, we applied harmonic constraints to the *C*_α_ atoms of the proteins, the phosphorus atoms of the lipid molecules, and the silicon and oxygen atoms of the silica pore, with spring constants of 50, 700, and 700 kJ/mol/nm^2^, respectively.

## Supporting Information Available

System characterization; insertion and relaxation of the biological membrane; sealing properties of the biohybrid nanopore; density distribution of ions; temporal evolution; representation of the AqpZ-incorporated lipid shell and POPC molecule; and radial distribution function (PDF)
Additional information about numerical simulation methods (ZIP)
Insertion of the AqpZ-incorporated lipid nanodisc into the solid-state nanopore (MP4)
Crossing of a water molecule through the AqpZ channel (MP4)
