# Supplementary material for: Biohybrid Membrane Formation by Directed Insertion of Aquaporin into a Solid-State Nanopore
Source: ACS Appl Mater Interfaces. 2022 Oct 16;14(42):48029–36. doi: 10.1021/acsami.2c14250 (PMC9614727; doi:10.1021/acsami.2c14250)
Supplement: Supplementary file 1 — am2c14250_si_001.pdf [file am2c14250_si_001.pdf]

Supporting Information

Biohybrid membrane formation by directed insertion of Aquaporin into a solid-state nanopore

François Sicard and A. Ozgur Yazaydin*
Department of Chemical Engineering, University College London, WC1E 7JE London, UK

*ozgur.yazaydin@ucl.ac.uk.

System characteristics

Aquaporin Z monomer. The crystal structure of *E. coli* AqpZ was obtained from the Protein Data Bank (PDB), entry 1RC2 [1]. The PDB file contains two protomers, from which protomer A was used to build the system simulated in this work. Missing side chains of residues Arg3, Glu31, Ser104, Arg230, and Asp231, as well as missing hydrogen atoms, were added using the programs Phyre2 [2] and VMD [3]. Titrable side chains were simulated in their default titration state, *i.e.* Glu and Asp residues with a negative charge, Lys and Arg residues with a positive charge, and all other side chains with zero charge. Therefore, the protein is electrically neutral and no counterions were needed to ensure the electroneutrality of the AqpZ monomer. Finally, the program DiANNA [4] was used to check for the presence of any disulfide bond.

AqpZ-incorporated lipid nanodisc. The program *Nanodisc Builder* [5] in CHARMM-GUI [6] was used to build the Aquaporin-incorporated lipid nanodisc. One AqpZ monomer was embedded in a 1-palmitoyl-2-oleoyl-sn-glycero-3-phosphocholine (POPC) lipid-nanodisc stabilized by the MSP1E2D1 membrane scaffold protein. The diameter and net charge of the corresponding nanodisc are 11.1 nm and -14e, respectively [7]. The number of POPC lipid molecules in the discoidal lipid bilayer is 230. The net charge of the MSP nanodisc was neutralized with 14 sodium ions.

Silica nanopore. The model solid-state nanopore was built from crystalline silica. The model of unit cell of cristobalite was obtained from the shared library of Materials Studio [8], which was periodically replicated in all directions. A regular silica slab was then carved out from the bulk cristobalite silica. Unsaturated silica atoms were removed from the surface of the slab and the non-bridging oxygen atoms were saturated with hydroxyl (-OH) groups. The structure was processed to saturate all valences, followed by energy minimization and molecular dynamics simulation to achieve structural stability. The volume of the equilibrated silica slab is $\sim (140 \times 140 \times 95)$ $\AA^3$. The resulting silica interface has a density of silanol groups of 6.4 OH/nm^2, within the range of experimental data [9]. Next, a 90 $\AA$ diameter cylindrical void was created in the center of the silica slab. Unsaturated silica atoms were removed from the interior surface of the pore and the non-bridging oxygen atoms were saturated with alkyl group $-C_nH_{2n+1}$, followed by energy minimization and molecular dynamics simulation to achieve structural stability. We considered different functionalizing group with $n = 1$, 2, and 3. The ideal silica membrane model was adopted in this work to study the effect of interaction between the biological membrane and the pore surface on sealing property.

Insertion and relaxation of the biological membrane in the solid state nanopore

To model the directed insertion of the biological membrane in to the model silica pore, a hydrostatic pressure difference of $\Delta P \sim 80$ MPa is created across the pore and is applied for 10 ns, while the position of the solid-state membrane was restrained. The value of ΔP was chosen to achieve the insertion of the nanodisc in a reasonably short simulation time while preserving the structure of AqpZ, as shown in Figure S1 with the evolution of the Cartesian backbone RMSD of the protein. The structural deformation of the lipid membrane after relaxation is quantitatively assessed with the measure of the elongation along the z-axis of the two hydrocarbon chains forming the hydrophobic tails of the lipid molecules, *i.e.* $C2 - C218$ and $C2 - C316$ defined in Figure S2(a), as a function of the lipid radial position, as shown in Figure S2(b) and (c), respectively. We observe the elongation of both hydrocarbon tails as the lipid molecules radially position away from the center of the protein, in agreement with the gauche-trans conformation described in the main text.

Sealing properties of the biohybrid nanopore

We studied the effect of the length of the alkyl group $-C_nH_{2n+1}$, which functionalizes the interior surface of the silica pore, on the sealing property of the biohybrid membrane. In particular, we considered methyl ($n = 1$), butyl ($n = 2$), and propyl ($n = 3$) groups. In Figure S3 is shown the schematic representation of the system after it relaxed for 1 ns, when the interior surface of the pore was functionalized with different alkyl groups. Unlike Figure S3 (c), where the interior surface of the pore is functionalized with propyl groups, we observed in Figures S3 (a) and (b) the presence of water molecules at the interface between the lipid molecules and the interior surface of the silica pore functionalized with methyl and butyl groups, respectively, which can be explained by the abscence of preferential interaction between the hydrophobic tail of the lipids and the short alkyl (methyl or butyl) groups.

Density distribution of ions in sea water environment

In Figure S4 is shown the density distribution of ions (sodium and chloride ions) in the simulation box after the system relaxed for 200 ns at constant temperature $T = 300$ K. The ion density in the AqpZ-incorporated lipid shell, i.e. $|z| \leq 1$, is exactly zero, indicating the efficiency of the membrane to play the role of separator. For $1 \leq |z| \leq 4$ we observe the progressive increase of the ion density, similar to the one observed for the water molecules (cf. main text). This profile is explained by the conformational organisation of the lipid bilayer in the nanopore due to preferential interaction with the hydrophobic surface. For $|z| \geq 4$, *i.e.* outside the solid-state nanopore, the system transitions abruptly to reach the bulk desired ion density.

ΔP	traj 1		traj 2		traj 3		Water flux (#/ns)	Crossing time (ns)
	Δt (ns)	# crossings	Δt (ns)	# crossings	Δt (ns)	# crossings		
10 MPa	24	12	24	1	24	17	0.42 ± 0.34	2.7 ± 1.1
25 MPa	24	14	24	6	24	18	0.53 ± 0.25	2.5 ± 1.0
50 MPa	24	14	24	27	24	41	1.14 ± 0.56	2.7 ± 1.9
75 MPa	24	55	24	34	24	48	1.90 ± 0.44	1.9 ± 0.8
100 MPa	24	58	24	64	24	60	2.52 ± 0.12	2.2 ± 1.1

TABLE I: Summary of the number of crossings, water flux and crossing time associated with the passage of the water molecules across the biohybrid membrane as a function of the hydrostatic pressure difference, ΔP. Uncertainties were determined by dividing the trajectories into three equal sections (traj 1, traj 2, and traj 3) of duration $\Delta t \sim 24$ ns, and calculating the standard error.

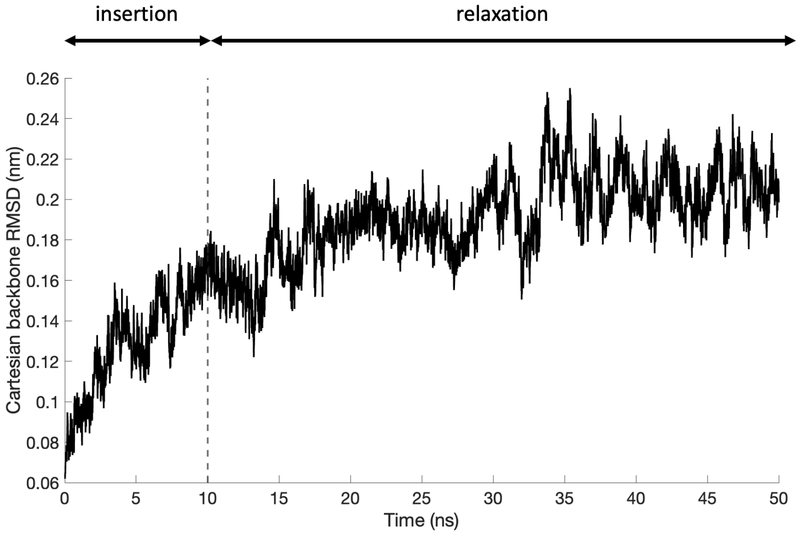

Figure S1: Temporal evolution of the Cartesian backbone root-mean-square deviation (RMSD) of the AqpZ monomer during the directed insertion of the biological membrane and the first 40 ns of relaxation.

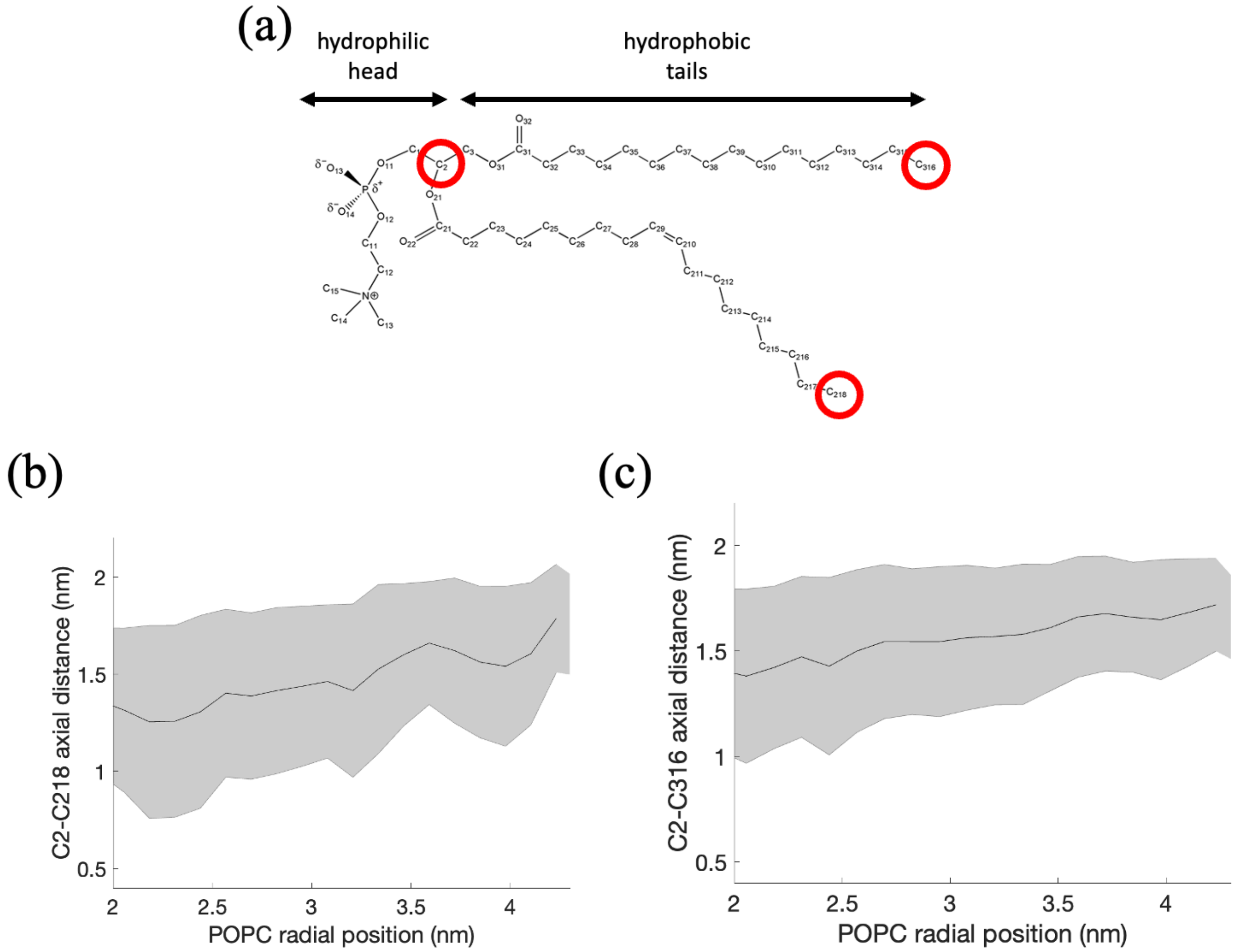

Figure S2: (a) Schematic representation of the POPC molecule. The carbon atoms $C2$, $C218$, and $C316$, used in the definition of the hydrocarbon tails of the POPC molecule, are circled in red for clarity. The distribution of the axial distance measure between the carbon atoms $C2 - C218$ and $C2 - C316$ is shown in panels (b) and (c), respectively. Uncertainties, defined as the standard error, are represented by the shaded area. We observe the elongation of both hydrocarbon tails as the lipid molecules radially position away from the center of the protein.

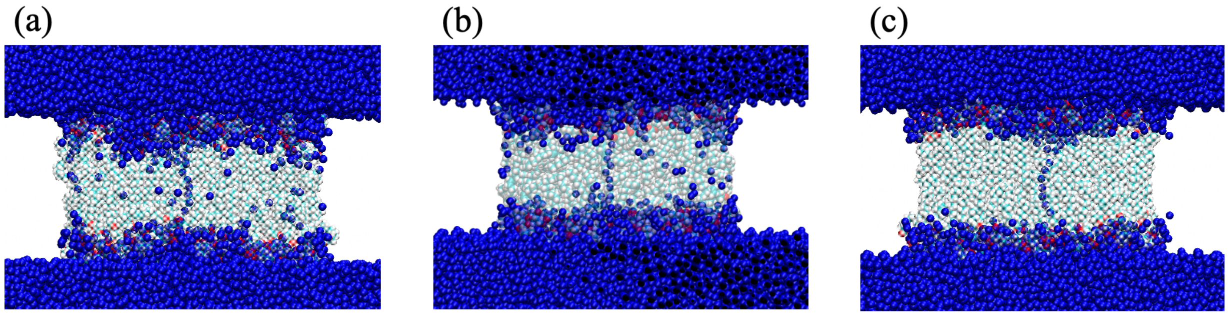

Figure S3: Schematic representation of the AqpZ-incorporated lipid shell inserted in the silica nanopore functionalized with the (a) methyl, (b) butyl, and (c) propyl group, after the system relaxed for 1 ns. Red, blue, white, gold, and cyan spheres represent oxygen, nitrogen, hydrogen, phosphate and carbon atoms in the lipid molecules, respectively. The oxygen atoms of the water molecules are shown in blue. The lipid molecules are shadowed and the silica nanopore and the AqpZ monomer are not shown for clarity. In addition to the water channel which connects both sides of the membrane, we observe the presence of water molecules at the interface between the lipid molecules and the interior surface of the silica pore functionalized with methyl and butyl groups, as shown in panels (a) and (b), respectively.

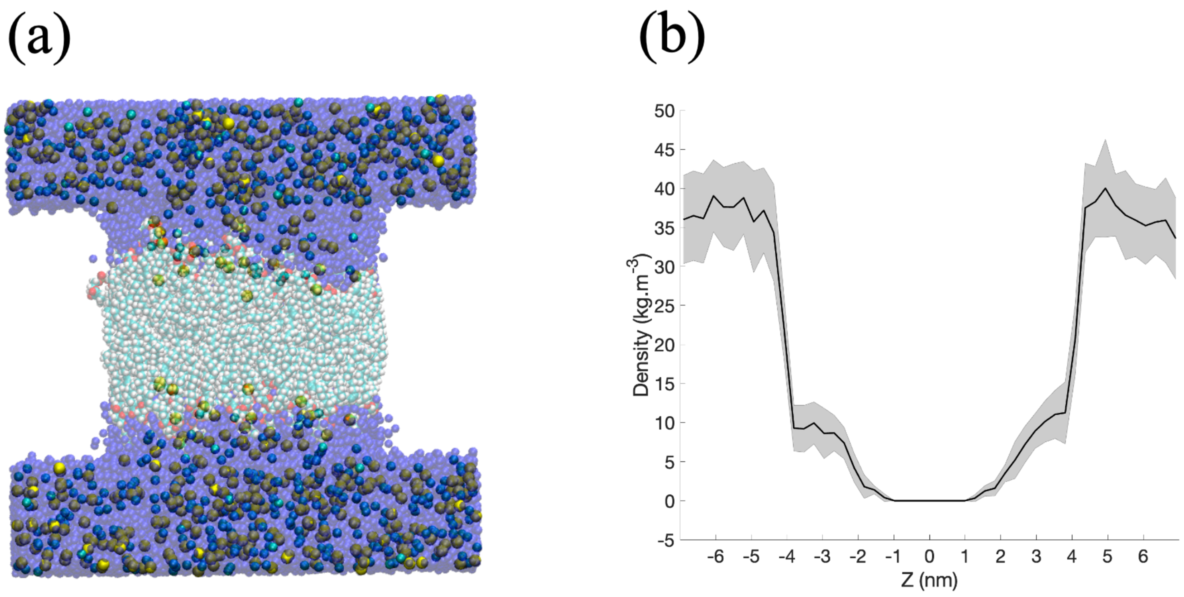

Figure S4: (a) Schematic representation of the system in sea saline water. The solid-state nanopore is not shown for clarity. The chloride and sodium ions are shown in green and yellow, respectively. Red, blue, white, gold, and cyan spheres represent oxygen, nitrogen, hydrogen, phosphate and carbon atoms in the lipid molecules, respectively. The oxygen atoms of the water molecules are shown in blue. (b) Density profile of ions (sodium and chloride ions) along the Z direction of the simulation box centered around the AqpZ monomer, measured after the system relaxed for 200 ns in sea water environment. Uncertainties, defined as the standard errors, are represented by the shaded area.

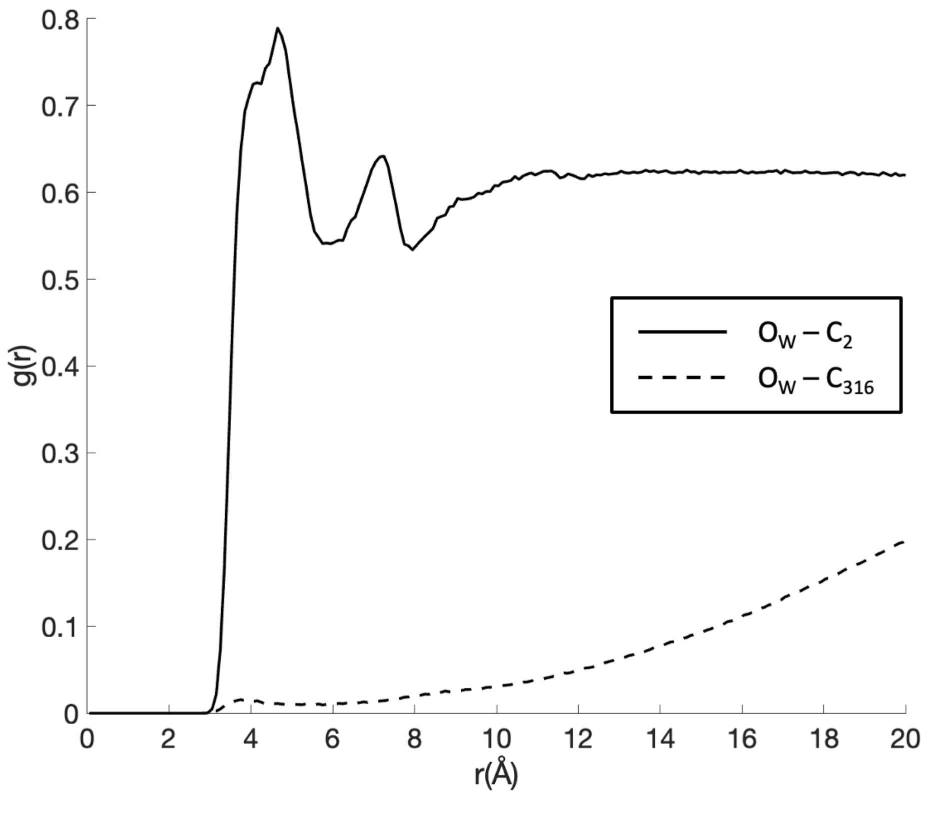

Figure S5: Radial distribution function, $g(r)$, of the water molecules (O_W) with respect to the carbon atoms C_2 and C_{316}, as defined in Figure S2, respectively.

[1] Savage, D.; Egea, P.; Robles-Colmenares, Y.; III, J. O.; Stroud, R. Architecture and Selectivity in Aquaporins: 2.5 ÅX-Ray Structure of Aquaporin Z. *PLoS Biol.* **2003**, *1*, e72.

[2] Kelley, L.; Mezulis, S.; Yates, C.; Wass, M.; Sternberg, M. The Phyre2 Web Portal for Protein Modeling, Prediction and Analysis. *Nat. Protoc.* **2015**, *10*, 845–858.

[3] Humphrey, W.; Dalke, A.; Schulten, K. VMD - Visual Molecular Dynamics. *J. Mol. Graph.* **1996**, *14*, 33–38.

[4] Ferré, F.; Clote, P. DiANNA: a Web Server for Disulfide Connectivity Prediction. *Nuc. Acids Res.* **2005**, *33*, 230–232.

[5] Qi, Y.; Lee, J.; Klauda, J.; Im, W. CHARMM-GUINanodisc Builder for Modeling and Simulation of Various Nanodisc Systems. *J. Comput. Chem.* **2019**, *40*, 893–899.

[6] Jo, S.; Kim, T.; Iyer, V.; Im, W. CHARMM-GUI: A Web-Based Graphical User Interface for CHARMM. *J. Comput. Chem.* **2008**, *11*, 1859–1865.

[7] Errasti-Murugarren, E.; Bartoccioni, P.; Palacín, M. Membrane Protein Stabilization Strategies for Structural and Functional Studies. *Membranes* **2021**, *11*, 155.

[8] BIOVIA, Dassault Systémes, U., San Diego Materials Studio.

[9] Emami, F.; Puddu, V.; Berry, R.; Varshney, V.; Patwardhan, S.; Perry, C.; Heinz, H. Force Field and a Surface Model Database for Silica to Simulate Interfacial Properties in Atomic Resolution. *Chem. Mater.* **2014**, *26*, 2647–2658.
